# The Dental Section of the International Congress

**Published:** 1885-12

**Authors:** 


### THE DENTAL SECTION OF THE INTERNATIONAL CONGRESS.

The dentists of the United States have watched with absorbing interest the various steps in the organization of the International Medical Congress of 1887. Remembering the signal success that attended the formation of the Section of Oral and Dental Surgery in the Congress of 1881, of which many of them were members, they were anxious that the dentists of America should have an opportunity to exhibit the progress that had here been made, and to demonstrate to the world the fact that legitimate dentistry was a science worthy the attention and encouragement of every other practitioner of the healing art. They received with great gratification the announcement that the next session of the Congress would be held in this country, and upon the formation of the Dental Section were ready to put forth every effort, and to secure for it, if possible, a yet more prosperous issue than had ever attended a convocation of dentists. They were prepared to accept the organization as announced, and had commenced preliminary work for securing a large attendance from this and other countries.

They understood that this was to be a World's Assembly of all that was truly representative in legitimate medicine, quite irrespective of society affiliations, or special vexed ethical questions. When, therefore, they became convinced, by the action of the American Medical Association at its meeting at New Orleans, that a single society had usurped the supreme control of that which was intended to be a council in which the medical profession of the whole world could meet upon equal grounds, they began to fear that the success of their Section was seriously prejudiced.

Following this came the announcement that the Section of Dental and Oral Surgery was, by the reorganized committee, abolished, and that dentists would not be welcomed to the Congress, upon the ground that such medical men as had devoted their lives to the consideration of diseases of the oral cavity were not engaged in the legitimate practice of any branch of the healing art. This annunciation, by those in control of the organization of the Congress of 1887, after the distinguished success of the Dental Section of that of 1881, and the entire harmony that had marked all the deliberations of that meeting, and in view of the high scientific status which the practice of dental surgery and dental medicine had attained in this country, was the source of the greatest surprise to the dentists of America, nor are they now prepared to believe that a specialty that had met with full recognition by the profession of England, is considered by the intelligence of the medical men of the United States entirely illegitimate.

When, on account of defections from the ranks of the supporters of the organization of the Congress of 1887, it became evident that its scientific success was jeopardized, and the aid of the dentists was desired, without a word of apology or explanation the Section was re-established. But they fear that this action was prompted by motives of policy, and not from a real change in the convictions of the committee. Under such circumstances, we do not believe that a proper regard for our own dignity or that of our confreres in other countries, permits us again to enter a meeting from which we have once been repulsed.

In another part of this number will be found the report of a conference of leading dentists, called together by the action of Prof. Taft, the President of the Section, and held in the City of Buffalo. There was a singular unanimity of sentiment concerning the duty of the dentists in this emergency. Prof. Taft acted as chairman, and himself expressed no decided opinion. Dr. Allport, who has, from the inception of the scheme for a Dental Section, been especially active in its advancement, expressed the belief that success was within our reach, and that all differences would be healed, but we think we are correct in saying that not one of those present believed that any decided steps should be taken, under existing circumstances, for the active organization of the Section. The most that was urged was that we should wait until the dissensions in the medical profession should be healed.

The resolution adopted provides for this. If the opposing medical factions unite, and the Congress promises to be something more than an enlarged session of the American Medical Association, we shall most heartily join with others in doing all possible to make the Dental Section one worthy the dentists of America. But we want no part in a rump Congress, and we have too high a regard for our calling to wish to see it take any part in a faction fight.

